# Treatment of relapsed or refractory Hodgkin’s lymphoma with lenalidomide combined with PD-1 monoclonal antibody: a case report and literature review

**DOI:** 10.3389/fonc.2025.1632039

**Published:** 2025-07-09

**Authors:** Hongjuan Dong, Rui Wang, Lu Cheng, Shirong Ma, Li Zhu, Jing Zhao, Guangxun Gao

**Affiliations:** ^1^ Department of Hematology, Xijing Hospital, Fourth Military Medical University, Xi’an, Shaanxi, China; ^2^ Department of Pathology, Xijing Hospital, Fourth Military Medical University, Xi’an, Shaanxi, China

**Keywords:** Hodgkin lymphoma, lenalidomide, PD-1 monoclonal antibodies, relapsed/refractory, case report

## Abstract

The current standard treatment for relapsed or refractory Hodgkin’s lymphoma involves salvage chemotherapy followed by high-dose chemotherapy and sequential autologous stem cell transplantation. However, for patients who cannot tolerate intense chemotherapy or do not meet the conditions for autologous stem cell transplantation, there is a need to explore new treatment options. We report a case of an elderly patient with multiple relapses of classical Hodgkin’s lymphoma who experienced repeated disease progression despite undergoing multiple lines of treatment including novel agents such as CD30 monoclonal antibody and PD-1 monoclonal antibody. The patient finally achieved sustained remission after receiving a combination therapy of lenalidomide and PD-1 monoclonal antibody. We hope to provide hematologists with a candidate treatment option for patients with relapsed or refractory Hodgkin’s lymphoma through this case report.

## 1 Introduction

Hodgkin’s lymphoma is a malignant tumor of the blood system originating from B-cells. Classic Hodgkin’s lymphoma (cHL) is one of the most common types of Hodgkin’s lymphoma, with a bimodal distribution in incidence, primarily affecting young adults and those over 55 years old. The typical pathological feature is the presence of Reed-Sternberg (R-S) cells set within a characteristic background of various types of reactive inflammatory cells (1). The prognosis for patients with cHL is generally favorable, with approximately 80% of young patients achieving cure after standard first-line treatment. Despite the high cure rate after initial treatment, approximately 5%-10% of cHL patients are resistant to initial therapy, and 10%-30% of patients relapse after achieving initial Complete Remission (CR), ultimately progressing to relapsed or refractory cHL (R/R cHL) (2).

For patients with cHL who respond to initial therapy but later relapse, salvage chemotherapy followed by high-dose chemotherapy and sequential autologous stem cell transplantation (ASCT) remains the most commonly used standard treatment. However, not all patients are suitable or can benefit from ASCT, and approximately 50% of those who undergo ASCT still experience relapse. Simultaneously, compared to younger patients, elderly patients receiving ASCT have an increased treatment-related mortality rate and generally lower event-free survival rates (2). To address this issue, several novel agents have emerged, such as CD30 monoclonal antibodies, Programmed Death Receptor 1(PD-1)/programmed death-ligand 1 (PD-L1) antibodies, and antibody-drug conjugates (ADCs), which have demonstrated efficacy as monotherapy or combination therapy, as well as in consolidation treatment after ASCT (3). Currently, the CD30-targeted ADCs brentuximab-vedotin (BV) has demonstrated significant efficacy in the initial induction therapy, post-transplant maintenance/consolidation therapy, and treatment of R/R HL, establishing itself as one of the standard treatment options for HL patients. The initial clinical trials of BV primarily enrolled post-transplant patients, revealing promising clinical outcomes. In a pivotal phase II trial evaluating BV in HL patients who failed ASCT, 75% of patients achieved clinical responses, with 33% attaining CR (4). Similarly, antibody-mediated blockade of PD-1 interaction with PD-L1 or PD-L2 has also yielded high clinical response rates in this patient population (5–7), supporting PD-1 blockade as the preferred treatment option for patients relapsing after ASCT or those ineligible for ASCT. Combination therapies, such as BV paired with two immune checkpoint inhibitors (nivolumab and ipilimumab), have shown encouraging efficacy in triple-regimen protocols (8). Other emerging strategies include chimeric antigen receptor (CAR) T-cell therapy, which, despite being in early-stage development, has demonstrated acceptable safety and notable clinical activity (9, 10). Despite these advances, there remains an unmet need for effective and tolerable treatment options for R/R HL patients, particularly those unsuitable for ASCT.

This report presents a case of an elderly male patient diagnosed with cHL (nodular sclerosis type). Following standard treatment, the patient experienced recurrence and was intolerant to intensive chemotherapy regimens. After undergoing various treatment strategies, including second-line chemotherapy, PD-1 monoclonal antibody therapy, and CD30 monoclonal antibody-targeted immunotherapy, the patient’s condition was transiently controlled but relapsed and progressed multiple times. However, sustained remission was achieved through the use of a combination therapy consisting of lenalidomide and a PD-1 monoclonal antibody.

## 2 Case report

The publication of this case report has obtained written informed consent from the patient.

**Figure 1:**
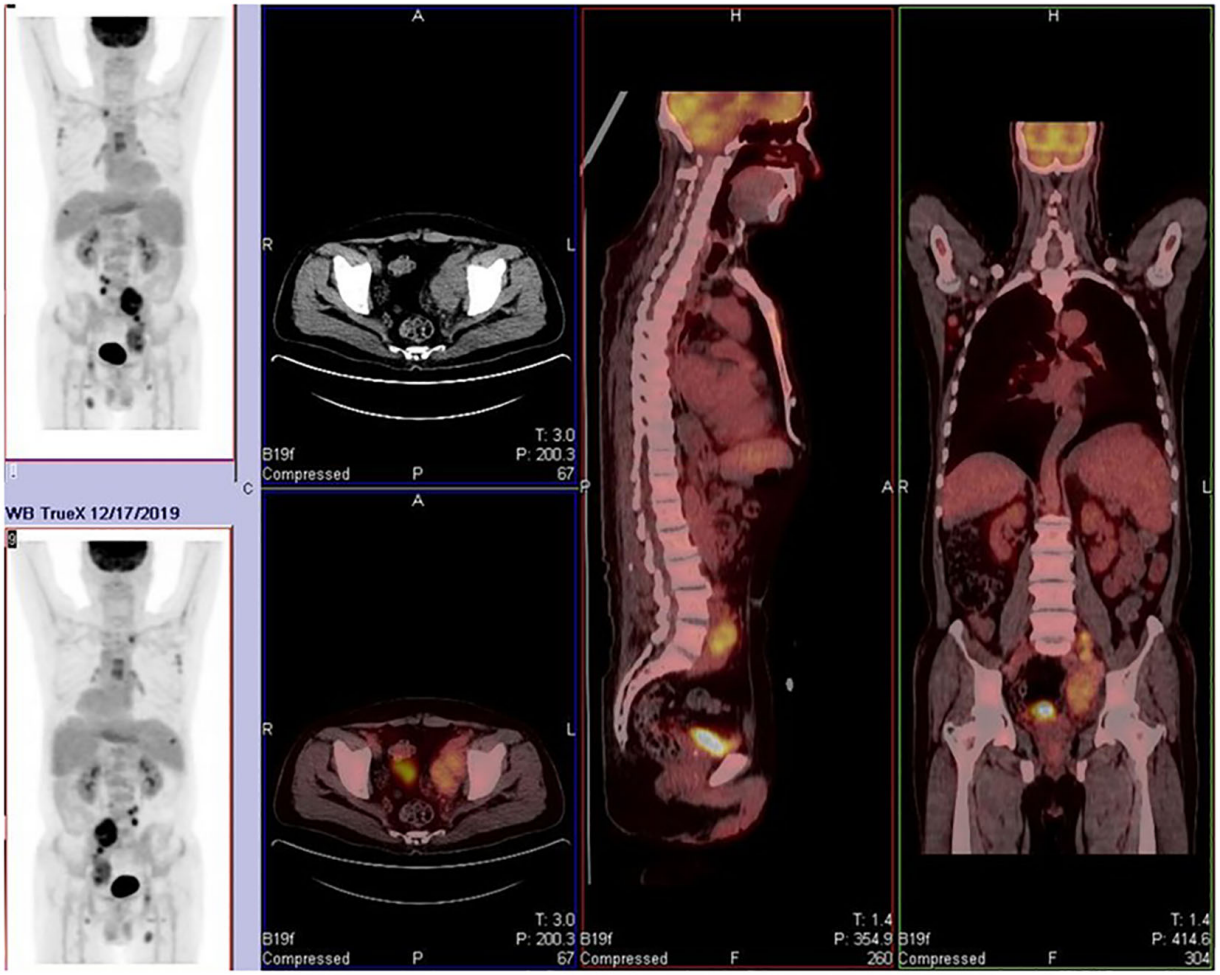
Widespread lymph node and skeletal involvement. Affected sites include bilateral cervical roots, axillary, mediastinal, hilar, retroperitoneal, and inguinal lymph nodes. Osteoblastic lesions were observed in multiple bones, including the humerus, clavicle, sternum, ribs, and acetabulum. These findings are consistent with Stage IV lymphoma.

The patient is a 68-year-old male. He was first admitted to the hospital in 2019 due to bilateral inguinal lymphadenopathy with intermittent fever. A lymph node excision biopsy was performed at another hospital, and our hospital’s pathological consultation revealed the disappearance of the normal lymph node structure with scattered R-S-like cells. Immunohistochemistry results showed Pax-5(+), CD20 and c-Myc (partially +), CD30 and CD15(+), CD21 (FDC disorder), CD3 (T cell +), ALK (ALK p80) (–), Ki-67 (approximately 25%), and EBER *in situ* hybridization (+). Combining morphological and immunohistochemical phenotypes, the diagnosis was classical Hodgkin’s lymphoma (nodular sclerosis type). Simultaneously, a PET/CT scan (**Figure 1**) was performed to refine the diagnosis according to the Ann Arbor staging system. The final diagnosis was classical Hodgkin’s lymphoma, stage IV B (nodular sclerosis type) with bone marrow involvement. The patient received three cycles of ABVD (doxorubicin, bleomycin, vinblastine, dacarbazine) chemotherapy from December 20, 2019, to April 7, 2020, and reported improvement in his symptomatic discomfort.

**Figure 2:**
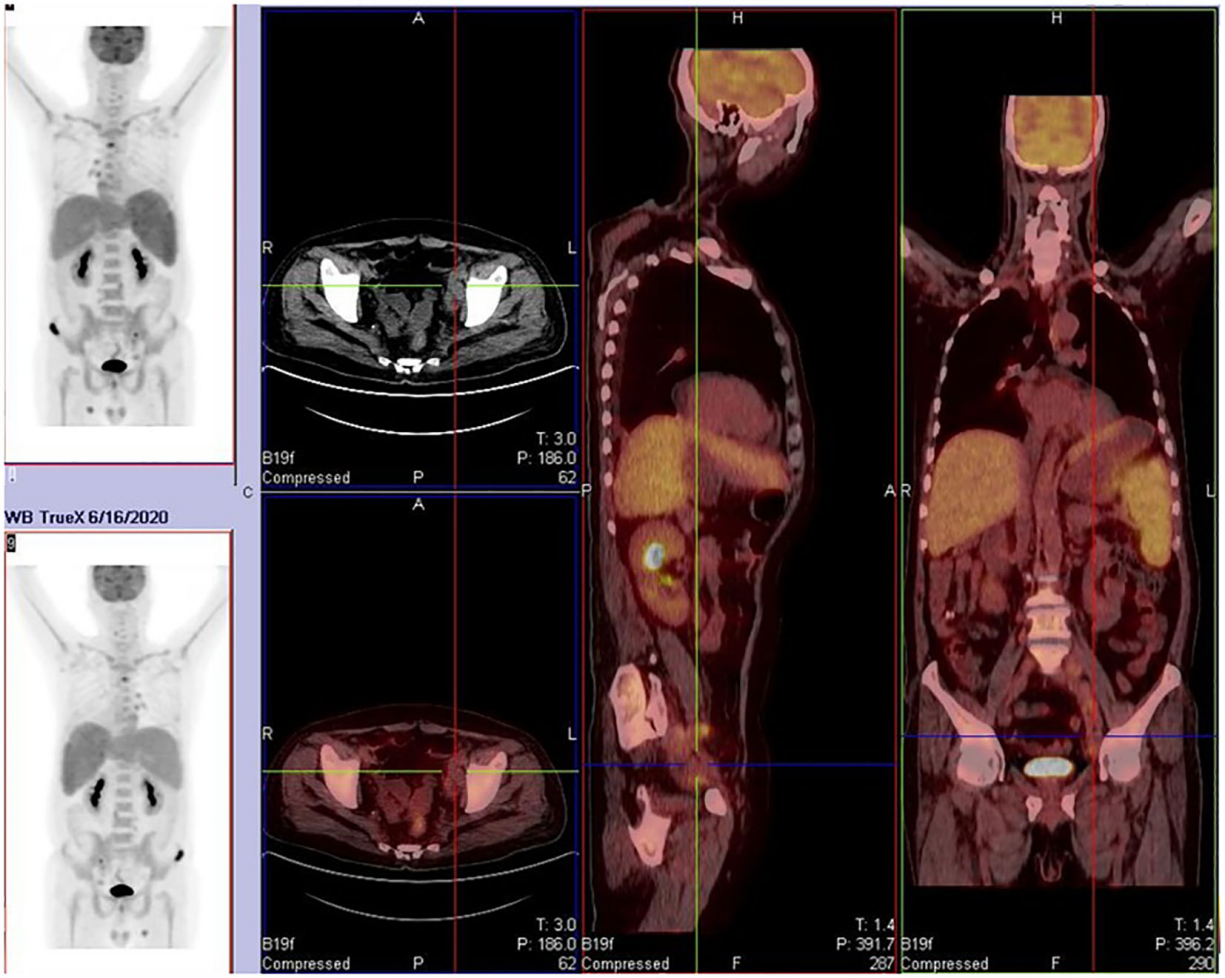
Partial lymph node regression with decreased glucose metabolism was noted, while some lymph nodes and pre-existing bone lesions remained metabolically active. New osteolytic lesions (e.g., clavicle, spine, pelvis) were observed, indicating disease progression.

**Figure 3:**
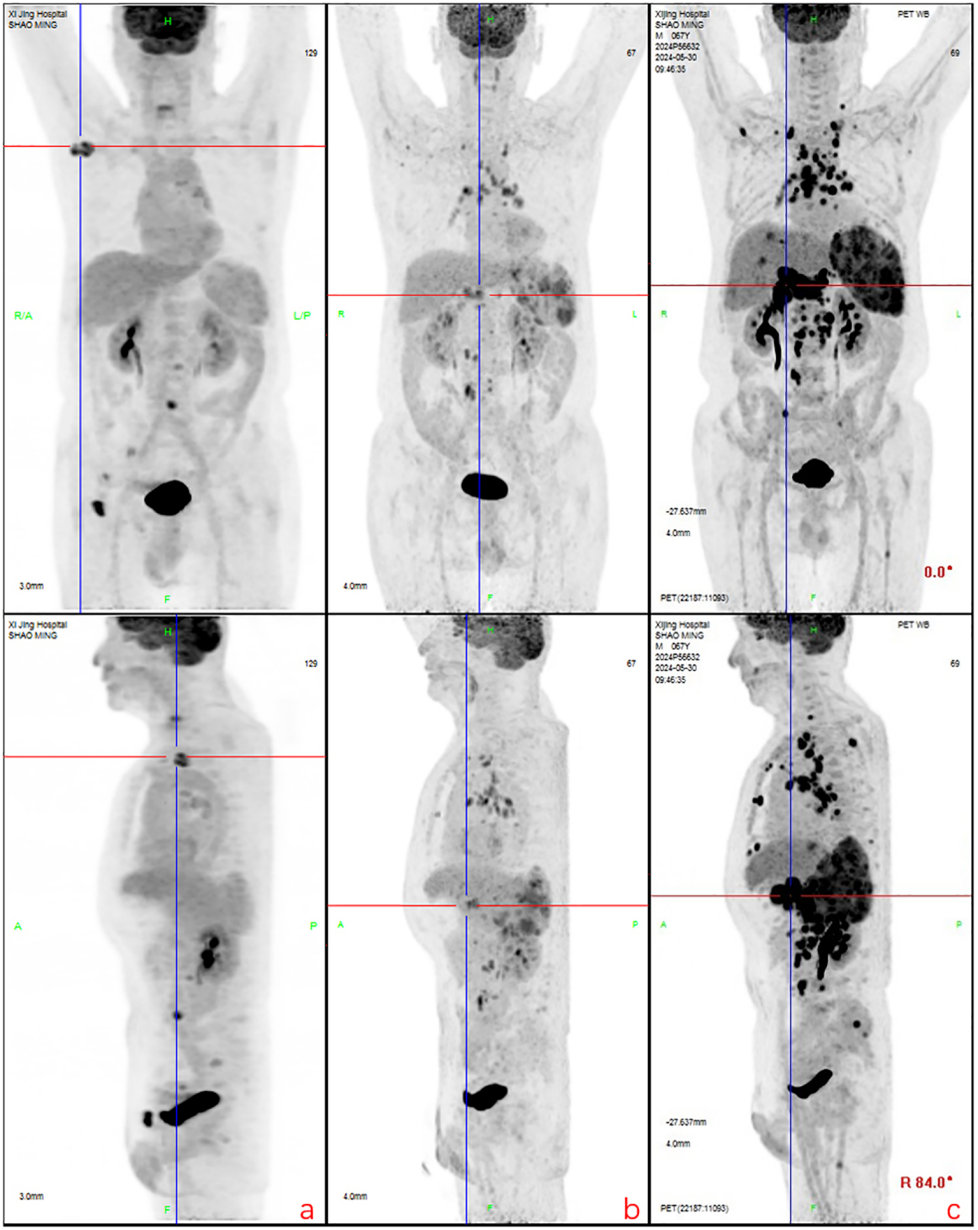
Partial lymph node shrinkage with reduced glucose metabolism was observed. New hypermetabolic lymph nodes were detected in the peripancreatic, internal mammary chain, and retroperitoneal regions. Recurrent metabolic activity was noted in prior bone lesions (e.g., iliac bone), along with new osseous infiltrations. The spleen exhibited persistent enlargement, and new hypermetabolic hepatic lesions (suggestive of lymphomatous infiltration) were identified, consistent with disease progression. **(a)**: Regression and metabolic resolution were observed in cervical root, paravertebral, and para-aortic lymph nodes. Most bone lesions showed metabolic resolution except for two residual iliac lesions. New hypermetabolic lymph nodes appeared in the right axillary, mediastinal, and left para-aortic regions. **(b)**: Right axillary and inguinal lymph nodes decreased in size, while mediastinal, hilar, and retroperitoneal lymph nodes exhibited reduced metabolism. New hypermetabolic lymph nodes were detected around the pancreatic head, internal mammary chain, and para-aortic region, accompanied by **splenomegaly with increased metabolic activity. **(c)**: Generalized progression was evident, with enlargement and increased metabolism in multiple lymph nodes (e.g., axillary, mediastinal, retroperitoneal). Bone lesions demonstrated recurrent metabolic activity, and previously inactive regions showed renewed hypermetabolism. The spleen continued to enlarge, and new hypermetabolic hepatic lesions indicated worsening visceral infiltration, confirming comprehensive disease progression.

On April 21st, 2020, the patient presented with fever again without apparent cause, reaching a maximum temperature of 39.6°C. The fever was predominantly afternoon-type and was not accompanied by other symptoms. Upon admission, infection indicators and lymphoma-related markers were assessed. The patient tested positive for PCT and G-experiment. Ultrasonography revealed an increase in spleen size compared to previous measurements, while lymph nodes had decreased in size. Based on these findings, it was considered that the fever was related to both infection and the primary disease. From May 10th, 2020, the patient was treated with caspofungin combined with cefoperazone-sulbactam for anti-infective therapy. Although the intervals between fever episodes became longer, and the peak temperature decreased, the patient still experienced persistent fever. Therefore, a second-line GDP (gemcitabine, cisplatin, dexamethasone) regimen was initiated on May 20th, 2020, for the treatment of lymphoma. After the first half of the chemotherapy cycle, the patient’s temperature normalized, but there was a decrease in three lineages of blood cells, with the lowest platelet count reaching 10×10E9/L, indicating grade four myelosuppression. Consequently, the second half of the chemotherapy cycle was postponed. On June 2nd, 2020, the patient developed fever again in the afternoon, with a temperature of up to 39.3°C, not accompanied by chills, cough, or other discomfort. Infection indicators were negative, and a whole-body PET/CT scan (**Figure 2**) suggested progression of the primary disease. The patient had Rh-negative blood, which is extremely rare, and the previous lowest platelet count had dropped to 10×10E9/L. Both the patient and their family members were extremely concerned about the risk of severe myelosuppression caused by intensive chemotherapy, coupled with the scarcity of Rh-negative blood, which would leave the patient in a state of extremely low platelet count and high risk of bleeding for a long time. Therefore, they refused intensive chemotherapy and bone marrow transplantation. On June 18th, 2020, the treatment was switched to PD-1 monoclonal antibody (Sintilimab). Since June 21st, 2020, the patient’s temperature has been normal, and no further fever episodes have occurred. Regular follow-up ultrasonography showed partial shrinkage of the originally enlarged lymph nodes, suggesting the effectiveness of the treatment. After 18 cycles of Sintilimab therapy, the patient underwent a whole-body PET/CT scan (**Figure 3a**) on October 12th, 2021 to evaluate the disease status. Considering the progression of the primary disease, the treatment was changed to BV for better disease control.

**Figure 4:**
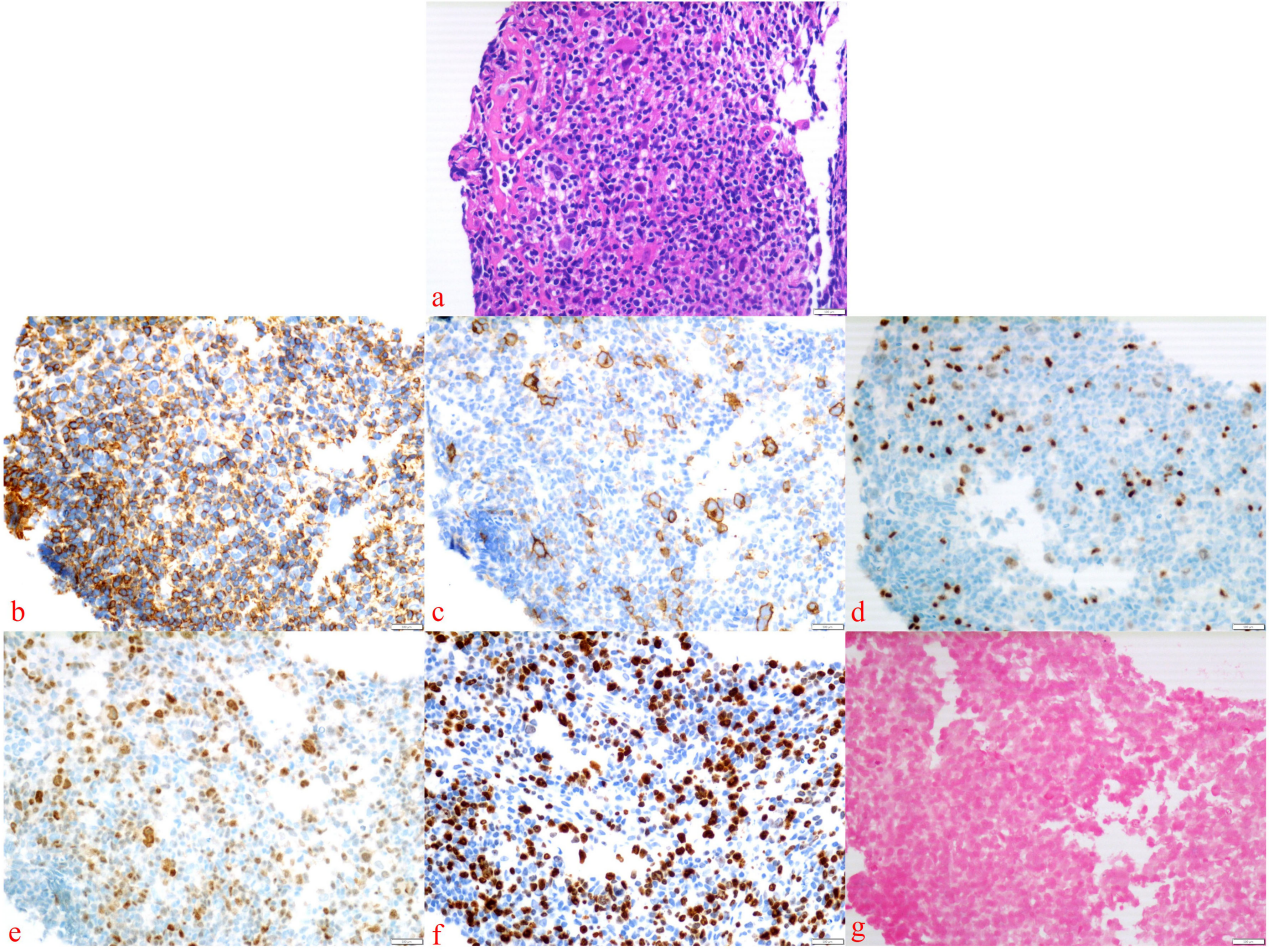
Abdominal enlarged lymph node biopsy was performed. The presence of Hodgkin’s cells was observed **(a)**, with the following immunohistochemical staining results: Bcl-6 (–), CD15 (–), CD20 (–), CD3 (–), LCA (–) **(b)**, CD30 (+) **(c)**, Pax-5 (weakly positive) **(d)**, MUM1 (+) **(e)**, Ki-67 (+) **(f)**, and EBER *in situ* hybridization (–) **(g)**.

After eight courses of BV treatment, the patient underwent a whole-body PET/CT scan (**Figure 3b**), which indicated local progression of the primary disease. The treatment regimen was then modified to a combination of CD30 monoclonal antibody and PD-1 monoclonal antibody therapy (BV + Tislelizumab). Following the completion of eight treatment courses, the patient presented again with fever, fatigue, night sweats, weight loss, and lymphadenopathy on May 28, 2024. A second abdominal enlarged lymph node puncture biopsy was performed, and the results showed significant lymph tissue hyperplasia with scattered atypical large cells (**Figure 4**). Simultaneously, a whole-body PET/CT scan indicated disease progression (**Figure 3c**). After communication with the patient and their family, the treatment plan was adjusted to include lenalidomide (25mg taken from day 1 to 21, followed by a one-week break) combined with tislelizumab (200mg, once every 3 weeks) starting from June 14, 2024. Following the initial switch to the treatment regimen comprising lenalidomide combined with PD-1 monoclonal antibody, the patient developed a grade 1 rash. Upon identification, immediate corticosteroid therapy was administered, leading to complete resolution of the rash. No further adverse reactions were observed during the treatment period. Currently, the patient has completed 10 cycles of treatment with lenalidomide combined with PD-1 monoclonal antibody. The patient’s fatigue and night sweats have resolved, weight has increased, no fever has occurred, and superficial lymph nodes and spleen have decreased in size compared to previous measurements, suggesting effective treatment. Since the onset of the disease, the patient has undergone five PET/CT scans. Currently, the patient reports no discomfort, and ultrasonography shows partial reduction in the size of superficial and deep enlarged lymph nodes and a decrease in spleen enlargement compared to previous measurements. However, due to economic reasons, the family has not opted for further PET/CT scans for evaluation.

## 3 Discussion

Here, we report a case of an elderly patient with recurrent cHL. At the initial diagnosis of Hodgkin’s lymphoma, our patient opted for the standard ABVD treatment. However, after completing three cycles of treatment, the patient experienced disease progression. Subsequently, a second-line GDP chemotherapy regimen was administered, but the patient developed severe myelosuppression and further disease progression. Sequential treatments with PD-1 monoclonal antibody, CD30 monoclonal antibody, and a combination of CD30 and PD-1 monoclonal antibodies achieved temporary disease control, but progression occurred again in each case. Finally, we adjusted the treatment plan to lenalidomide combined with PD-1 monoclonal antibody, resulting in the resolution of B symptoms and a reduction in the size of lymph nodes and spleen. Currently, the patient has undergone 10 cycles of treatment, with sustained stability in his condition, allowing him to return to a normal life.

The initial treatment plan for patients with cHL should be determined by the stage of diagnosis and risk factors. The primary chemotherapy regimen acceptable for cHL is the ABVD protocol, while the BEACOPP (bleomycin, etoposide, doxorubicin, cyclophosphamide, vincristine, procarbazine, prednisone) regimen is commonly employed as an alternative therapeutic option interchangeable with ABVD. For patients who are refractory to standard treatment, the selection of a therapeutic approach must be highly cautious. The preferred option is salvage chemotherapy followed by high-dose chemotherapy combined with ASCT. However, more than half of these patients may still experience recurrence. The anti-CD30 ADCs brentuximab-vedotin has been established as the standard treatment regimen for this patient population. Other potential therapeutic strategies include CAR-T cell therapy and novel ADCs. Although CAR-T cell therapy remains in early-phase development, it has demonstrated favorable safety profiles and significant clinical activity (10). Additionally, AFM13, the world’s first CD30/CD16A bispecific innate cell engager, selectively eliminates CD30+ tumor cells by recruiting and activating natural killer (NK) cells. These studies suggest that CAR-T therapy and bispecific antibodies may emerge as promising future treatment options for relapsed patients (11). However, the feasibility of these therapies remains limited in the present case. Therefore, more effective, better-tolerated, and more accessible treatment regimens must be explored for this patient.

The pathological hallmark of cHL is the presence of malignant R-S cells within a potent inflammatory milieu (12). This is attributed to the secretion of various cytokines and chemokines by R-S cells, altering the cellular composition and function of the surrounding microenvironment. The microenvironment adjacent to R-S cells plays a pivotal role in the pathogenesis of cHL (13). Lenalidomide, as an immunomodulatory drug, can influence the clinical response in treating cHL by modulating chemokine levels (14). Additionally, its direct antitumor activity, inhibition of tumor cell proliferation, anti-angiogenic and immunostimulatory effects, as well as its ability to stimulate Treg cell-mediated antitumor responses, may also contribute to the treatment of cHL (15–17). Lenalidomide is currently approved for use in mantle cell lymphoma, follicular lymphoma, marginal zone lymphoma, and diffuse large B-cell lymphoma (18). Our choice of lenalidomide for this patient was primarily based on the inflammatory cell infiltration and multiple immunomodulatory targets present in the microenvironment of cHL. Lenalidomide can enhance tumor recognition, i.e., immune response, to improve therapeutic efficacy. A study by Fehniger et al. on patients with R/R cHL demonstrated a median overall survival (OS) of 23.7 months in the study population. They also measured CCL17/TARC levels in the patient cohort and found that CCL17 levels declined in patients responding to lenalidomide treatment (19). Ma et al. reported a clinical benefit rate of 47% with continuous low-dose lenalidomide administration in patients with relapsed Hodgkin’s lymphoma after ASCT. In heavily pretreated patients with R/R cHL, daily continuous administration of low-dose lenalidomide was associated with higher disease control rates and reduced toxicity (20). These data suggest that patients with R/R cHL can benefit from lenalidomide treatment. Considering the mechanism of action of lenalidomide and its proven clinical efficacy, we decided to incorporate lenalidomide into the current patient’s treatment regimen, ultimately yielding exciting results.

Within the microenvironment of cHL, besides the copious cytokines and chemokines secreted by R-S cells, there exists a mixture of immune and stromal cells, among which T-cells predominate. The majority of these T-cells express Programmed Death Receptor 1(PD-1), a co-inhibitory receptor belonging to the CD28 superfamily. Through interaction with its corresponding ligands (PD-L1 and PD-L2) on Antigen Presenting Cells (APCs), PD-1 inhibits SHP-2 signaling within T-cells, thereby suppressing cytokine production and T-cell proliferation. This attenuation of T-cell response promotes T-cell tolerance. Studies have also revealed that PD-L1 can be expressed on tumor cells to evade anti-tumor immune responses. In cHL, R-S cells often exhibit copy number alterations of PD-L1 and PD-L2 on chromosome 9p24.1, leading to the overexpression of PD-1 ligands on tumor cells. The activation of the PD-1/PD-L1 pathway weakens the T-cell-mediated anti-tumor response, thus promoting a “tumor-friendly” microenvironment cHL (21). Nivolumab, a fully human monoclonal IgG4 antibody targeting PD-1, was first discovered by Younes et al. to elicit frequent response in cHL patients who had failed ASCT and B-V treatment. These responses were mostly sustained during the reported follow-up period, and the safety profile was acceptable (22). In multiple retrospective studies, Nivolumab has demonstrated a high response rate (23). Although Nivolumab exhibits clinically significant activity, only a few patients achieved CR. Tislelizumab, a humanized IgG4 monoclonal antibody, binds to the extracellular domain of human PD-1 with high specificity and affinity, blocking the binding of PD-L1 and PD-L2. It minimizes binding to FcγR on macrophages, thereby eliminating antibody-dependent phagocytosis (24). A clinical study by Song et al. found that the overall response rate of R/R cHL patients treated with Tislelizumab was 87.1%, with a CR rate of 67.1%. The duration of response was durable, with a median duration of response of 31.3 months and a median Progression-Free Survival (PFS) of 31.5 months (25). Based on the above research data, we chose to incorporate Tislelizumab into our treatment regimen. After patients received our combined treatment plan, B symptoms disappeared, lymph nodes and spleen shrank, and the disease was effectively controlled. Currently, patients can live a normal life. This demonstrates that the combination of Tislelizumab and lenalidomide may become a new treatment option for patients with R/R cHL.

The selection of a combined regimen of lenalidomide and PD-1 monoclonal antibody for this patient was inspired by a study conducted by Güllü Görgün and their team. In patients with multiple myeloma, the expression level of PD-1 on the surface of tumor cells is significantly elevated. Blocking the PD-1/PD-L1 pathway using PD-1 monoclonal antibodies can significantly enhance the killing effect of autologous T cells, NK cells, and monocytes/macrophages on multiple myeloma cells. Furthermore, the study found that the immunomodulatory drug lenalidomide not only directly affects multiple myeloma cells but also reduces PD-1 expression in various effector cells, including CD4T cells, CD8T cells, NK cells, and NKT cells. Additionally, it decreases PD-L1 expression in multiple myeloma cells, MDSCs (Myeloid-Derived Suppressor Cells), and monocytes/macrophages. Their research further confirms that immune checkpoint signaling plays a critical role in promoting tumor growth and immunosuppression in the bone marrow microenvironment of patients with multiple myeloma. Blocking PD-1 or PD-L1, either alone or in combination, can induce an anti-multiple myeloma immune response, which can be further enhanced by lenalidomide (26). Additionally, the humanized anti-CD19 monoclonal antibody tafasitamab in combination with lenalidomide has been approved by international treatment guidelines for the management of relapsed/refractory diffuse large B-cell lymphoma (R/R DLBCL) in patients ineligible for ASCT. These findings provided valuable insights for the therapeutic approach in this case (27). Inspired by these studies, we hypothesized that the combination of monoclonal antibody and lenalidomide, which has shown promising results in MM and DLBCL, might also exhibit corresponding effects in Hodgkin’s lymphoma. As anticipated, the patient’s condition showed significant improvement.

HL is characterized by the presence of a scant population of tumor cells (R-S cells) surrounded by a prominent but dysfunctional immune-reactive infiltrate (lymphocytes, plasma cells, macrophages, etc.), which plays a critical role in disease pathogenesis. The immunomodulatory drug lenalidomide not only increases the number of NK cells and enhances their activity and NK-mediated cytotoxicity but also lowers the activation threshold of NK cells (28, 29). Meanwhile, PD-1 monoclonal antibodies block the PD-1/PD-L1 pathway, significantly augmenting the tumoricidal effects of autologous T lymphocytes, NK cells, and monocytes/macrophages (1). Therefore, the combination of these two agents may exert synergistic therapeutic effects by enhancing NK cell- and T cell-mediated cytotoxicity, thereby amplifying the immune response in patients. Our next step will involve ex vivo studies to further elucidate the precise mechanisms underlying this combination therapy in cHL. Additionally, a study by Rebecca et al. demonstrated that mutations in noncoding regions of indolent lymphomas, through super-enhancer redirection, may drive transformation into aggressive lymphomas with increased malignancy (30). Given that our reported case exhibited a prolonged disease course with multiple relapses, we cannot exclude the possibility of noncoding region mutations contributing to treatment resistance and disease recurrence. We plan to systematically collect tumor tissue samples from such patients to further investigate the mechanisms underlying their refractory/relapsed disease.

To sum up, the results have proven that our patient’s general condition has greatly improved after using the combined regimen, achieving clinical remission in a short period and maintaining stable disease progression. However, due to economic reasons, disease evaluation following the treatment regimen adjustment was performed using ultrasonography rather than PET/CT. Although ultrasonography can assess the degree of lymphadenopathy, PET/CT imaging remains an essential tool for initial staging and treatment response evaluation in HL patients (31). We will further communicate with the patient and family members to recommend a follow-up PET/CT scan for accurate therapeutic assessment. Given the recurrent nature of this case and the multiple lines of treatment administered, long-term follow-up is imperative. To date, the patient has completed 10 treatment cycles with significant improvement in quality of life. We will continue to monitor their therapeutic outcomes and follow-up data.

## 4 Conclusion

Our clinical experience has initially demonstrated that the combination therapy of lenalidomide and PD-1 monoclonal antibody exhibits certain efficacy in patients with relapsed classical Hodgkin’s lymphoma who have previously undergone multiple lines of treatment and cannot tolerate the toxicity of chemotherapy. This regimen is well-tolerated without significant toxicity. Our patient had undergone various treatment regimens including novel agents in the early stages, yet the disease continued to relapse and progress. However, after one course of treatment with lenalidomide combined with PD-1 monoclonal antibody, the patient’s B symptoms disappeared, and there was a reduction in lymph node and spleen size. Currently, the patient has been on this treatment regimen for 10 months, with sustained disease stability and a return to a quality normal life. Additionally, while the combination therapy achieved good efficacy in this reported case, further clinical trials are still needed to determine whether this approach is suitable for all patients with R/R cHL.

## 5 Limitation

First, due to economic reasons, follow-up with PET/CT could not be performed after lenalidomide PD-1 combination therapy. Secondly, this report is a single-case study, documenting only one patient whose disease was controlled with lenalidomide and PD-1. Given the absence of a control group, drawing broadly applicable conclusions remains challenging. In future studies, we plan to collect similar cases to facilitate large-sample clinical research.

Explicitly stated compliance with patient consent norms, as required for biomedical publications.

## Data Availability

The original contributions presented in the study are included in the article/supplementary material. Further inquiries can be directed to the corresponding author.
